# Aspartame Safety as a Food Sweetener and Related Health Hazards

**DOI:** 10.3390/nu15163627

**Published:** 2023-08-18

**Authors:** Shurooq Asaad Abdulameer Shaher, Dan Florin Mihailescu, Bogdan Amuzescu

**Affiliations:** 1 Department of Biophysics and Physiology, Faculty of Biology, University of Bucharest, 005095 Bucharest, Romania; drshuruq73@gmail.com (S.A.A.S.); d.f.mihailescu@gmail.com (D.F.M.); 2 Department of Medical Laboratories, Babylon Technical Institute, Al-Furat Al-Awsat Technical University, Najaf 54001, Iraq

**Keywords:** aspartame, artificial sweetener, excitotoxicity, neuropsychiatric symptoms, reactive oxygen species, carcinogenic risk

## Abstract

Aspartame is the methyl-ester of the aspartate-phenylalanine dipeptide. Over time, it has become a very popular artificial sweetener. However, since its approval by the main food safety agencies, several concerns have been raised related to neuropsychiatric effects and neurotoxicity due to its ability to activate glutamate receptors, as well as carcinogenic risks due to the increased production of reactive oxygen species. Within this review, we critically evaluate reports concerning the safety of aspartame. Some studies evidenced subtle mood and behavioral changes upon daily high-dose intake below the admitted limit. Epidemiology studies also evidenced associations between daily aspartame intake and a higher predisposition for malignant diseases, like non-Hodgkin lymphomas and multiple myelomas, particularly in males, but an association by chance still could not be excluded. While the debate over the carcinogenic risk of aspartame is ongoing, it is clear that its use may pose some dangers in peculiar cases, such as patients with seizures or other neurological diseases; it should be totally forbidden for patients with phenylketonuria, and reduced doses or complete avoidance are advisable during pregnancy. It would be also highly desirable for every product containing aspartame to clearly indicate on the label the exact amount of the substance and some risk warnings.

## 1. Introduction

**Figure 1:**
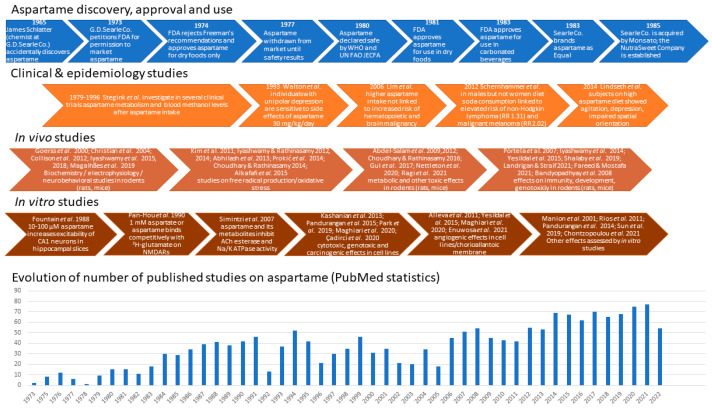
Main steps related to aspartame discovery, approval, development, and use, along with some of the clinical trials, *in vivo* and *in vitro* studies evidencing its biological effects (more details in Table A1), and the dynamics of publications describing them [7,8,9,10,11,12,13,14,15,16,17,18,19,20,21,22,23,24,25,26,27,28,29,30,31,32,33,34,35,36,37,38,39,40,41,42,43,44,45,46,47,48,49,50,51,52,53,54,55,56,57,58,59].

Due to decreased sugar production throughout the two world wars as well as an increased prevalence of nutrition disorders, particularly diabetes mellitus, in post-war industrialized societies, artificial sweeteners, also known as non-nutritive sweeteners, gained popularity [1]. Some non-nutritive, low-calorie sweeteners provide a similar taste while bringing 200–300 times fewer calories than sugar [2]. Saccharin, discovered and used since 1879, was widely regarded at the time as a sugar substitute. James Schlatter, while doing biochemical synthesis experiments with Robert Mazur, accidentally discovered aspartame in 1965, and his employer G.D. Searle immediately started testing the substance in the hope of producing and commercializing it on a wide scale [3,4] (Figure 1). Aspartame is the methyl ester of the dipeptide formed by L-aspartic acid and L-phenylalanine [5]. It has been found to be 188 times sweeter than sugar while having the same calorie contents per weight unit [6].

In 1973, D. Searle requested from the Food and Drug Administration (FDA) an initial clearance for aspartame. The FDA stated that the maximum daily dosage of aspartame for humans should be 50 mg/kg body weight/day in the United States; in Europe, a maximal acceptable daily intake (ADI) of 40 mg/kg body weight/day was adopted [60]. However, some chronic exposure and carcinogenesis studies [61,62] found—even in female rats exposed to relatively low doses of aspartame (4 or 20 mg/kg body weight/day)—exposures that are close to the current ADI in the European Union (40 mg/kg body weight/day) bring an increased incidence of malignant tumors.

By 1983, the Equal^®^ brand of aspartame sugar replacement ruled the tabletop non-nutritive sweetener industry. Aspartame appealed to diabetics because its dipeptide composition did not require insulin to be metabolized, and it featured a better sweet taste devoid of bitterness and presumably fewer side effects compared to earlier sugar substitutes, such as saccharin and cyclamate. Large soft drinks companies, such as Coca-Cola^®^ and Royal Crown^®^, declared they would combine two artificial sweeteners, e.g., aspartame and acesulfame K, in their low-calorie diet soda products in order to minimize the side effects of each of them [1,7,63], a guideline followed so far. One year after full approval was obtained, in 1984, NutraSweet^®^ quickly developed into a very lucrative division of G.D. Searle, and the number of customers grew less affected by safety concerns [64]. Thus, nowadays, aspartame is a common component in over 6000 food products and beverages.

Aspartame is present in soft drinks, dessert mixes, yogurt, chewable multivitamins, and morning cereals. Millions of people throughout the world ingest it because it is also present in 600 different types of medicines [65]. The production of low-calorie beverages, which are widely consumed by youngsters and pregnant people, is a crucial use in the United States [66,67]. Although some early studies estimated that the amount of methanol intake resulting from diet soda drinking in a hot environment could reach 250 mg/day or 32 times the Environmental Protection Agency’s daily limit suggestion [68], a more realistic estimate places the methanol intake resulting from daily aspartame consumption in the highest 90% as being 25 times lower than the maximal safe level of methanol intake of 7.1–8.4 mg/kg/day established by FDA, and much lower than the methanol intake resulting from other natural sources, such as pectin, fruits, vegetables, and alcoholic beverages [69]. A substantial body of the literature shows that young animals are more vulnerable than older animals to a variety of chemical and physical carcinogens, particularly during the prenatal period [70]. A re-examination of histopathology data from large groups of animals fed on aspartame-containing diets within studies performed at the Ramazzini Institute in Bologna confirmed that aspartame exposure during pregnancy raises the risk of cancer in rodent offspring [8]. Additional research into associations between aspartame and conditions, like brain tumors, brain lesions, and lymphoma, has also been advised by several researchers [61,71]. The Food Additives and Nutrient Sources Added to Food Panel evaluated the potential risks of aspartame for pregnant women by assessing the plasma concentrations of the breakdown product phenylalanine following the consumption of aspartame-containing products [60,72].

**Figure 2:**
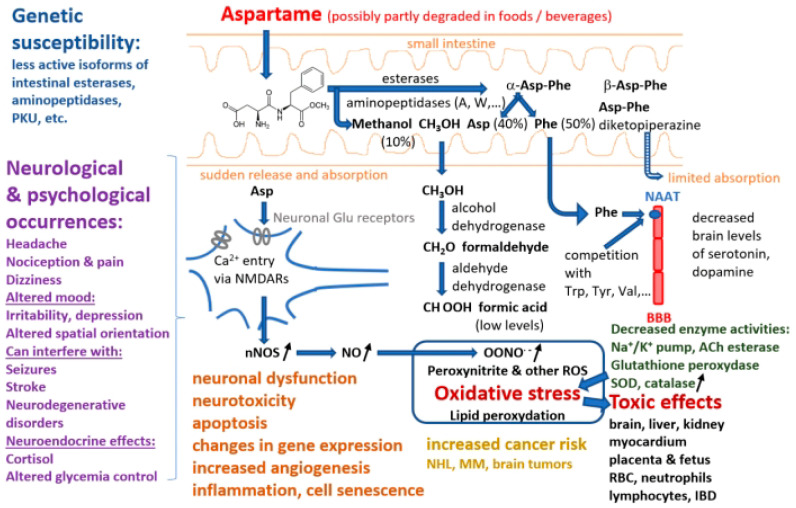
Summary of pathways involved in aspartame decomposition, kinetics, metabolism, potential adverse effects at cell level, side effects, and relationships with different disorders (abbreviations: PKU—phenylketonuria, NAAT—large neutral amino acids transporter, BBB—blood–brain barrier, NMDARs—N-methyl-D-aspartate receptors, nNOS—neuronal nitric oxide synthase, NO—nitric oxide, ROS—reactive oxygen species, ACh—acetylcholine, SOD—superoxide dismutase, RBC—red blood cell, IBD—inflammatory bowel diseases, NHL—non-Hodgkin lymphoma, MM—multiple myeloma).

As consumption is on the rise among the general population, it is essential to create awareness regarding the usage of this artificial sweetener. Therefore, the aim of this review is to summarize the available literature regarding the potential health risks of aspartame (Table A1), including carcinogenesis hazards, with a special emphasis on neurological and psychological effects, integrating evidence gathered in clinical trials, in vivo and *in vitro* studies and proposing some molecular targets, pathways, and mechanisms that may explain these effects (Figure 2).

## 2. Chemical Structure, Digestion, and Metabolism

Aspartame is the methyl ester of a dipeptide composed of a hydrophilic and a hydrophobic amino acid residue, aspartic acid (Asp) and phenylalanine (Phe), respectively, giving it some unique qualities [73,74]. Aspartame in purified solid form is a white crystalline powder that may be stored at temperatures between 30 and 80 °C and is extremely stable under dry conditions [75]. At room temperature, its aqueous solution has a half-life of approximately 300 days and reaches the highest stability at a pH of 4.3, which is common for diet sodas. The peptide bonds are hydrolyzed in certain conditions, such as high temperature or basic pH [1].

Aspartame stability in soft drinks has been studied intensively. Thus, it was found that after 50 weeks storage at room temperature of a diet soda, 20% of its aspartame content was de-esterified to α-Asp-Phe, 15% was converted to β-Asp-Phe and β-aspartame, and another 20% was converted into a cyclic dipeptide known as aspartame diketopiperazine (3-carboxyl-methyl-6 benzyl-2.5 diketo-piperazine) [75,76]. Aspartame produces methanol by hydrolysis in highly acidic or alkaline environments. The peptide bonds are also hydrolyzed in more extreme circumstances, releasing the free amino acids. Upon consumption, aspartame is split by several digestive enzymes, such as esterases and peptidases, into a number of chemical components, including aspartic acid, phenylalanine, and methanol, the latter being further decomposed into formaldehyde and formic acid [77]. Studies with human and pig intestinal microvilli preparations and specific inhibitors showed that aminopeptidases A and W are the most active in decomposing the α-Asp-Phe dipeptide [78]. Another pioneering study proved that Asp-Phe is hydrolyzed by three of four brush border peptidases and by a cytosolic peptidase different from the seven known isoforms [5]. Similarly, amino acids and dipeptide intestinal absorption studies [79] showed that, although dipeptide absorption mechanisms are present, particularly in the jejunum and ileum [80], α-aspartame is almost entirely decomposed in the intestinal lumen and passes into circulation as Asp (40%), Phe (50%), and methanol (10%) [69,76]. The rates of intestinal absorption of β-Asp-Phe and aspartame diketopiperazine are small [76]. The group of Lewis Stegink was particularly active and performed a number of clinical studies on adults, children, and infants, involving acute (single-dose or repeated doses over less than 1 day) or prolonged (e.g., daily for 13 consecutive weeks) intake of aspartame doses, sometimes higher than the ADI (up to 200 mg/kg body weight) (Table A1) to assess the pharmacokinetics and demonstrate the lack of toxicity of aspartame decomposition products [9,10,11,12,13,14,15,16,81], except for subjects with genetic traits resulting in low plasma α-Asp-Phe hydrolase activity [17]. However, other clinical studies reached different conclusions, showing adverse effects of aspartame, particularly in subjects with neurological or psychiatric conditions, such as migraines [82,83], other headaches [84,85], or unipolar depression [18]. Part of these differences may result from the fact that aspartame was administered in some studies, e.g., [18], as powder included in enteric-soluble capsules, which can release very high concentrations of aspartame over limited areas of the intestinal mucosa, in contrast to administration in a pre-dissolved form in water or beverages.

While phenylalanine is turned into tyrosine and phenylethylamine, and methanol is converted into formaldehyde, which then undergoes an oxidation reaction to formic acid, aspartic acid is converted to alanine and oxaloacetate [86]. Each of these compounds is metabolized according to a natural metabolic route in the same manner as those originating from foods and other dietary sources. As demonstrated in animals, methanol from aspartame enters the portal circulation and is promptly converted by alcohol dehydrogenase to formaldehyde, which is further transformed into formate by aldehyde dehydrogenase [65]. Early pharmacokinetics and metabolism studies in humans have shown that upon acute ingestion of 50 mg/kg aspartame, blood methanol levels increased to 0.34 ± 0.12 mg/dL (mean ± SEM, *n* = 6) in adults 30–90 min after intake, and to 0.30 ± 0.10 mg/dL in infants; higher aspartame doses produced proportionally higher peak blood methanol levels [9,10]. However, several researchers have pointed out that methanol levels resulting from aspartame intake are several times smaller than those produced by consumption of other common foods and drinks, like fruit or vegetable juices and fermented distilled beverages, due to enzyme-driven breakdown of methoxyl groups of polysaccharides, such as pectin [9,69]. Therefore, aspartame side effects are more likely due to the two amino acids released by its decomposition, phenylalanine, and aspartate. Thus, a clinical study on children fed with aspartame 34 mg/kg/day for two weeks proved increased phenyalanine and tyrosine plasma levels compared to a placebo group [87].

The increased phenylalanine concentrations are linked to lower levels of catecholamines, serotonin, and dopamine [88]. Phenylalanine is a large neutral amino acid that competes with other amino acids for binding on the large neutral amino acid transporter [89]. Phenylalanine released from aspartame may theoretically mediate neurologic effects since it has neurotoxic potential and influences the production of monoamine neurotransmitters. When pentylenetetrazole, an epileptogenic drug, is administered to mice after aspartame administration, the frequency of seizures that follow is increased [90]. This is because aspartame causes plasma phenylalanine levels to rise more than those of tyrosine (which likely happens after any aspartame dose in humans). Phenylalanine prevents dopamine release in the striatum, whereas valine, which competes with phenylalanine for passage across the blood–brain barrier, can alleviate its proepileptogenic effect [91]. The reduced levels of dopamine and serotonin are a result of the excess phenylalanine blocking the transport of crucial amino acids to the brain. In addition to being employed in protein synthesis, phenylalanine can also be converted into the highly concentrated phenylpyruvic acid in phenylketonuria patients [19,92]. By competing for neutral amino acid transporters, phenylalanine can directly affect the entry of other critical amino acids into the CNS. As a result, it indirectly influences neurotransmitter deficiencies that result in functional problems [93].

On the other hand, aspartate, the carboxylate anion of aspartic acid, undergoes transamination in enterocytes to become oxaloacetate before reaching the portal circulation [69]. The urea cycle and gluconeogenesis can be affected by the body’s conversion of oxaloacetate and aspartate [2]. Aspartate and other related amino acids, such as asparagine, glutamate, and glutamine, did not significantly change their plasma levels in healthy people after taking aspartame doses of 34–50 mg/kg [14]. Aspartic acid residues are frequently found in proteins. The body may convert aspartic acid into the neurotransmitter glutamate, which at very high levels, can have harmful effects on the nervous system. In addition, high doses of aspartate can directly activate N-methyl-D-aspartate (NMDA) receptors, exerting excitotoxicity and other central nervous system adverse effects. However, the European Food Safety Authority’s experts did not see any evidence of neurotoxicity associated with aspartame and therefore concluded that aspartic acid derived from aspartame does not raise any safety concerns for consumers [60].

## 3. Mechanisms of Toxicity of Aspartame Metabolism Products

The fundamental tenet of toxicology is that all substances are harmful at some concentration. As a result, it is not surprising that aspartame or its components have negative effects on sensitive animal species when consumed at very high doses. Upon testing the effects of various aspartame doses on blood levels of aspartate, phenylalanine, and methanol, several studies proved that these levels were well below those associated with adverse effects in animal species, raising the important question of whether aspartame ingestion is potentially harmful to humans during normal use or abuse, in spite of the fact that the dietary exposure of consumers to these compounds is higher than that resulting from aspartame intake [12]. Although FDA and other regulatory agencies have established permissible daily intake guidelines for aspartame ingestion, there are many questions about its safety today.

Frequent high-dose aspartame intake may have nephrotoxic effects. Thus, according to experimental data from different animal species, long-term consumption of aspartame caused a dose-dependent increased production of free radicals in renal tissues as well as kidney injury, as proved by a search of several literature databases for publications on the adverse effects of aspartame on the kidney function from 1980 to 2016 [94]. Additionally, recent cohort studies showed a link between excessive aspartame use and an elevated risk for cardiovascular disorders [95]. The administration of aspartame caused oxidative stress and markedly reduced the activity of antioxidant enzymes, such as superoxide dismutase, catalase, glutathione peroxidase, and glutathione reductase in both rat liver and renal tissues [96]. Increased pro-oxidant levels, such as reactive oxygen and nitrogen species (ROS/RNS), or decreased antioxidant levels, which could cause cell malfunction and disintegration, are indicators of oxidative stress [97].

Several in vivo and *in vitro* studies revealed altered scavenging mechanisms, increased lipid peroxydation, and enhanced generation of ROS/RNS in the erythrocytes or serum of aspartame-treated animals [20,98,99] or in human neuroblastoma cells [100]. The imbalance in ROS/RNS neutralization induced by aspartame can affect neutrophil adhesion, the phagocytic index, as well as antibody titers and soluble immune complexes in phagocytic and immune system cells, including neutrophils and lymphocytes [99]. A cohort study proved that a high aspartame intake during pregnancy increased the risk of developing asthma and allergic rhinitis in offspring [101].

Other studies have shown that aspartame use may increase the chance of developing cancer. Thus, multiple epidemiology follow-up studies on large cohorts have revealed higher incidences of different cancers among high-dose chronic aspartame users [21,102,103]. High doses of aspartame (15–30 mM) were found to be cytotoxic *in vitro* on a human colorectal carcinoma cell line, also promoting chorioallantoic membrane angiogenesis *in ovo* and having a mild irritating potential at vascular level [22]. In both *in vitro* and *in vivo* settings, aspartame had a stimulatory effect on angiogenesis [23,24,25]. Low levels of aspartame administration (less than 40 mg/kg/day) were linked to an increase in oxidative stress in the spinal cord [26]. Similar effects of oxidative stress may occur in immune system cells, such as different lymphocyte subsets, altering innate and adaptive immunity, and increased cortisol levels, which may result in supplementary immunosuppression favoring the proliferation of malignant cells [99,104]. These findings shed new light on aspartame’s involvement in the development of cancer, but further research is required to fully understand this phenomenon. Although a systematic meta-analysis of the literature did not retrieve, on average, a positive association between aspartame consumption and the occurrence of cancer, this analysis was limited to clinical data, excluding a large number of animal studies [105]; other epidemiology studies on large cohorts found increased odds ratios for the association of aspartame consumption with non-Hodgkin lymphomas and multiple myelomas in male subjects [21]. In a more recent study, Guercio et al. found that patients with stage III colon cancer who consumed more artificially sweetened beverages had significantly lower rates of cancer recurrence and mortality [106].

Aspartame at low doses (up to 170 µM) significantly altered the mRNA expression of apoptotic genes in HeLa cells, up-regulating the expression of the antiapoptotic gene Bcl-2 while down-regulating the expression of the tumor suppressor gene p53 and the apoptotic gene Bax [27]. A recent study on human umbilical vein endothelial cells challenged with doses between 0.01 and 1 mM for 1–4 days likewise showed that aspartame at low concentrations exerted no cytotoxic effects [107]. However, higher doses of aspartame (1–20 mM) significantly inhibited cell growth and induced apoptosis of HeLa cells upon incubation for 24–48 h [108]. Another *in vitro* experiment conducted on endothelial cells and fibroblasts revealed that aspartame (up to 100 µM) increased the production of ROS linked to the cytotoxic effect, raised the level of the inflammatory mediator IL-6, and had a pro-angiogenic effect by inducing the production of regenerative cytokines and activating the mitogen-activated protein kinase (MAPK) pathway [23]. Animal studies of aspartame administration during pregnancy evidenced reduced placental, maternal, and fetal weight and umbilical cord length [28], as well as the rupture of interhemal membranes of the placenta, lysis of trophoblast cells, and increased vascular endothelial growth factor (VEGF) staining [29].

## 4. Neurological and Cytotoxic Effects by Activation of NMDA and Other Glutamate Receptors by Aspartame or Its Metabolites

Glutamate represents the main excitatory neurotransmitter in the central nervous system. Glutamate receptors are divided into the following two groups: metabotropic glutamate receptors (mGluRs), with seven transmembrane α-helical segments accommodating the ligand molecule at the center, similar to rhodopsin, and ionotropic glutamate receptors (iGluRs), tetrameric ligand-gated ion channels with large extracellular domains featuring multiple ligand-binding sites and four transmembrane α-helical segments per subunit. The three types of ionotropic glutamate receptors—NMDA, AMPA, and kainate receptors—are distinguished by varying ion selectivity, activating agents, and pharmacological agonists and inhibitors [109]. The N-methyl-D-aspartate receptors (NMDAR) are crucial molecular components of learning and memory via the complex phenomenon of long-term potentiation (LTP), which involves receptor phosphorylation triggered by calcium influx upon repeated stimulation [110]. However, several pathological conditions, such as ischemic stroke or neurodegenerative diseases, may lead to excitotoxicity, consisting of excessive synaptic glutamate release and NMDAR overactivation with massive Ca^2+^ inflow, resulting in neuronal cell death [111]. The same author pointed out that the developing human brain is exposed to excitotoxic compounds, such as those present in foods, to a much larger extent compared to the adult brain due to an immature blood–brain barrier [112]. Therefore, it seemed logical to express similar concerns over the use of aspartame since the compound itself and its decomposition product aspartate may effectively activate NMDARs [113], in addition to neurotransmitter imbalances caused by aspartate and phenylalanine [88,91].

Early *in vitro* assays revealed that aspartame, as well as L-aspartate, may directly act on the NMDA glutamate recognition sites in brain synaptic membranes because it significantly changed the affinities of l-[^3^H]-glutamate binding without changing the *V*_max_ of binding [30]. Further, Ca^2+^ inflow via activated NMDARs can trigger calmodulin-dependent activation of different tyrosine kinases and neuronal nitric oxide synthase (nNOS), and the increased NO production would result in higher levels of reactive oxygen and nitrogen species. Indeed, numerous in vivo studies evidenced markers of oxidative stress in animals on an aspartame diet compared to control groups in the brain and other tissues, such as the liver, kidney, and blood cells, including erythrocytes, neutrophils, and lymphocytes (Table A1). These markers are represented by the increased levels of superoxide, peroxide, and lipid peroxidation, decreased levels of reduced glutathione, glutathione reductase, and nitrite, and increased activity of free radicals scavenging enzymes like superoxide dismutase and catalase [20,26,31,32,33,34,35,114]. Free radicals produced in large amounts can damage membranes by peroxidation of unsaturated fatty acids in the phospholipids that make up the bilayer [97].

Some experimental studies found an increased Na^+^/K^+^ ATPase activity in membrane fractions from the midbrain of aspartame-fed rats [36], while other studies found a decreased activity in folate-deficient rats on aspartame diet [19,26] or in rat hippocampus homogenates or pure Na^+^/K^+^ ATPase incubated with aspartame metabolites [37]. Furthermore, aspartame-fed folate-deficient rats showed, in different brain regions, reduced levels of phosphorylated NMDAR1 subunits, increased iNOS and nNOS expression, and NO production [19]. The central nervous system can express all three isoforms of nitric oxide synthase (Ca^2+^-sensitive nNOS and eNOS, and Ca^2+^-independent iNOS), which produce nitric oxide (NO), further converted into the powerful free radical peroxynitrite [115]. When the body’s capacity to neutralize and eliminate ROS is surpassed, increasing oxidative stress is a potential threat [116]. This mechanism possibly underlies neuronal damage in multiple brain regions, as evidenced by several animal studies regarding the intake of aspartame [19,35,38,117] or L-aspartate [118]. In aspartame-exposed rats with early-life exposure to NMDAR antagonists, significant drug/diet interactions were reflected in glucocentric and behavioral measures. This suggests a potential role for early NMDAR interactions in aspartame-induced behavioral impairments and altered glucose homeostasis [63]. Nitric oxide/cGMP/glutamate release could be modulated by aspartame or aspartate stimulation of NMDA receptors along the sensory pathways, affecting reactivity to pain. This hypothesis is in agreement with the presence of functional NMDARs in approximately 32% of trigeminal primary sensory neurons in rats [119,120]. Aspartame would be an effective analgesic if combined with a calcium channel blocker or NOS inhibitor [121].

These multiple biochemical effects of aspartame and its metabolites on the nervous system may explain the changes in behavioral tests recorded in different in vivo studies for aspartame-fed animals relative to control groups. One common finding was the alteration of spatial orientation resulting in longer escape latencies in maze tests, such as the Morris water maze or Y-maze, upon intake of aspartame [19,39,63] or impaired flexibility of spatial memory for aspartate [122]. Another important effect is increased anxiety, explaining immobility, diminished ambulation and grooming [38], poorer performance in open field tests [63] and elevated maze-plus tests [40], reduced aggression, lengthened intervals between attacks, reduced number of bites per session [41], increased latency to reward [36], and decreased latency in passive avoidance tests for aspartate-fed mice [118]. Similar phenomena consisting of signs of depression and poor spatial orientation, without effects on working memory, were evidenced in an outstanding clinical trial on healthy young student volunteers fed for 8 days on a high-aspartame diet containing 25 mg/kg/day [42]. Other behavioral changes found in clinical trials were difficulty sitting still and paying attention for a preschool child with low plasma α-Asp-Phe hydrolase activity on aspartame diet [17], depression, insomnia, trouble remembering, and irritability for patients with unipolar depression given aspartame 30 mg/kg/day [18], as well as migraine and other types of headache, a common occurrence [82,83,84,85]. Several comprehensive reviews on neuropsychiatric effects related to aspartame consumption are available [69,72,88,89,91,93,104,113,123,124,125].

## 5. Conclusions

Aspartame is an additive used to sweeten a variety of beverages and foods, such as desserts, cakes, chewing gum, yogurt, low-calorie and weight-control products, and even drugs for oral administration. Its presence in foods can be indicated either by name or by its code E951. Following ingestion, aspartame breaks down in the gut into the following three constituents: aspartic acid, phenylalanine, and methanol. These components are also naturally present in other foods, including fruits and vegetables, and for foods containing aspartame, they are processed by the body in the same way as those derived from other dietary sources. Following a detailed and methodical analysis, European Food Safety Authority [60] experts concluded that aspartame and its breakdown products are safe for human consumption at current levels of exposure. The current ADI is considered to be safe for the general population, although some clinical [42] or *in vivo* animal studies [38] suggested neurobehavioral effects upon daily aspartame intake below or at ADI.

Questions have primarily been raised about the early experimental animal studies used to evaluate the safety of aspartame. Some subsequent studies concluded that there is sufficient scientific evidence to confirm that aspartame is generally safe for human consumption up to the maximally recommended daily intake doses. However, its use may pose health risks for certain individuals, like patients with seizures or other neurological conditions; it is strictly forbidden for patients with phenylketonuria and should be restricted if not completely eliminated during pregnancy. It is also highly advisable that each aspartame-containing product lists explicitly the exact amount of aspartame on its label. The association between high-dose aspartame usage and increased risk of developing cancers, such as brain tumors or non-Hodgkin lymphomas, is still highly controversial and under investigation and seems to be confirmed by some recent cohort studies.

## Data Availability

No new data were created or analyzed in this study. Data sharing is not applicable to this article.

## Appendix A

**Table A1:** Synopsis of epidemiology, clinical, in vivo, and *in vitro* studies exploring different effects and health hazards of aspartame.

Study	Method	Experimental Groups	Main Results
**EPIDEMIOLOGY STUDIES**			
Lim et al., 2006 [43]	Clinical cohort prospective study to assess the risk of hematopoietic cancers or malignant gliomas associated with aspartame consumption	285,079 men and 188,905 women aged 50–71 years with aspartame consumption assessed via a self-administered food frequency questionnaire, followed up for 5 years (1995–2000).	1888 hematological malignancies and 315 malignant gliomas were identified over the 5-year period; higher aspartame intake was not linked to an increased risk of hematological cancer (RR 0.98) or glioma (RR 0.73) in either men or women.
Schernhammer et al., 2012 [21]	Longitudinal prospective cohort study	1324 non-Hodgkin lymphomas (NHL), 285 multiple myelomas (MM), and 339 leukemias were isolated from two large patient cohorts that were studied for more than 20 years; participants were divided into habitual diet soda drinkers (1 daily serving) and non-consumers.	In males, but not in women, diet soda consumption was linked to an elevated risk of NHL (RR 1.31), and MM (RR 2.02). Men who drank more soda had a higher risk of developing NHL (RR 1.66). A coincidental association cannot be ruled out.
Maslova et al., 2013 [101]	Prospective longitudinal cohort study	60,466 women enrolled during pregnancy in the prospective longitudinal Danish National Birth Cohort between 1996 and 2003; validated food frequency questionnaire administered at gestation week 25; child asthma evaluated at 18 months.	Children of artificially sweetened carbonated drinks consumers were more likely to have asthma (OR 1.30, 95%CI: 1.01–1.66) and take specific medication (OR 1.13, 95%CI: 0.98–1.29), as well as self-reported allergic rhinitis (OR 1.31, 95%CI: 0.98–1.74) during the first 7 years of follow-up.
Bassett et al., 2020 [102]	Prospective cohort study	41,513 subjects aged 27–76 years at recruitment, of which 6404 were excluded due to pre-existing cancer, angina/heart attack, and diabetes, placement in top/bottom 1% of energy consumption or missing data.	Over 19 years of follow-up, 4789 of the 35,109 selected participants developed cancers not related to obesity; subjects with high intake of artificially sweetened drinks had a significant hazard ratio of cancer development compared to control (HR 1.23; 95%CI: 1.02–1.48; *p*-trend 0.006).
Debras et al., 2022a [103]	Longitudinal follow-up cohort study	NutriNet-Santé web-based cohort included 128,343 volunteers aged >18, of which 102,865 participants were selected and monitored over a median follow-up time of 7.8 years via daily 24 h dietary records.	High consumers of total artificial sweeteners had higher overall risk of cancers (HR 1.13, *p*-trend 0.002); for high-dose aspartame consumers HR 1.15, *p* = 0.002; higher risks were detected for breast (HR 1.22, *p* = 0.036) and obesity-related cancers (HR 1.15, *p* = 0.026).
Debras et al., 2022b [95]	Longitudinal follow-up cohort study	NutriNet-Santé web-based cohort included 103,388 selected participants, classified at baseline for diet, health, height and weight, lifestyle and socio-demographic data, physical activity, were monitored for dietary records and with biannual health questionnaires over the period 2009–2021.	Total artificial sweeteners (HR 1.09, *p* = 0.03) and aspartame (HR 1.03, *p* = 0.49) were associated with cardiovascular diseases, coronary heart diseases and cerebrovascular diseases; aspartame intake was associated with cerebrovascular events (HR 1.17, *p* = 0.02).
**CLINICAL STUDIES**			
Stegink et al., 1979 [14]	Randomized cross-over design	An overdose of aspartame (100 mg/kg body weight) was delivered to 6 fasting adult volunteers (3 men and 3 women) in both solution and slurry form.	Plasma aspartate increased from 0.16 ± 0.05 (mean ± SD) to 0.43 ± 0.23 µM (solution) or 5.8 µM (slurry form), phenylalanine from 5 to 20.3 ± 2.05 or 26.0 ± 18.9 µM, methanol increased to 1.16 ± 0.47 or 1.27 ± 0.48 mg/dL at 60–90 min after loading, far below toxic levels.
Stegink et al., 1981 [10]	Randomized cross-over design	30 healthy normal adult participants (15 men and 15 women) received different doses of aspartame *p*.*o*.: 12 subjects (6 male, 6 female) 34 mg/kg, and 6 subjects in each group were given 100, 150, and 200 mg/kg.	No differences on ophthalmologic examinations performed before and after aspartame loading, and no alterations in blood chemistry profile 24 h after aspartame ingestion.
Stegink et al., 1983 [9]	Clinical study of blood methanol levels in one-year-old infants administered graded doses of aspartame	24 infants aged between 8 and 14 months were tested in 3 stages: 10 subjects received aspartame 34 mg/kg, subsequently 6 subjects received 50 mg/kg, and 8 subjects 100 mg/kg body weight.	At 34 mg/kg blood methanol levels were below limit of detection (0.35 mg/dL), at 50 mg/kg 0.30 ± 0.10 mg/dL, at 100 mg/kg 1.02 ± 0.28 mg/kg, non-toxic and similar to those observed in normal adults.
Stegink et al., 1987 [12]	Balanced Latin square design to test effects of co-ingestion of monosodium L-glutamate with aspartame	12 healthy normal adult participants (6 males, 6 females) were administered with the following three distinct soup/beverage meals: meal A with no aspartame (APM) or monosodium L-glutamate (MSG); meal B with 50 mg MSG per kg body weight; meal C with 50 mg MSG and 34 mg APM/kg body weight.	Plasma glutamate levels increased significantly after meals B and C, and aspartate levels after meal C; plasma Glu + Asp mean levels increased from baseline (5.64 ± 2.62 µM) to 23.1 ± 7.29 µM (meal B) or 26.8 ± 9.74 µM (meal C).
Koehler and Glaros 1988 [82]	Controlled double-blind randomized cross-over study on the frequency and intensity of migraine	11 subjects with migraine history were exposed for 13 weeks to either 1200 mg aspartame/day or placebo and then switched regime after a wash-out period.	Aspartame ingestion by migraineurs caused a significant increase in headache frequency (*p* = 0.0144).
Schiffman et al., 1987 [84]	Double-blind cross-over clinical trial	40 subjects with history of headache/related neurological symptoms within 24 h after aspartame intake were challenged with 30 mg/kg aspartame or placebo.	The incidence of headache after aspartame ingestion (35%) or placebo (40%) were not different (*p* = 0.5); the subjects with headache had lower plasma concentrations of epinephrine (*p* < 0.0002) and norepinephrine (*p* < 0.02).
Stegink et al., 1988 [13]	Two-stage clinical trial in a standard cross-over design	8 healthy normal adult subjects (4 males, 4 females), were given in stage 1, 3 servings of unsweetened beverage, and in stage 2, 3 servings of beverage providing aspartame 10 mg/kg body weight each.	Addition of aspartame had no effect on plasma aspartate levels, and increased phenylalanine levels by 1.64–2.05 µM above baseline (5.09 ± 0.82 µM), not exceeding normal postprandial values.
Stegink et al., 1989 [16]	Balanced cross-over design	6 normal healthy adult subjects (3 male, 3 female) were given 8 beverage servings at 1 h intervals, unsweetened or providing 600 mg aspartame per serving.	Plasma phenylalanine levels increased by 1.41–2.35 µM above baseline 30 min after ingestion of aspartame-containing drinks and reached steady-state after 4–5 servings.
Walton et al., 1993 [18]	Cross-over design	Initially designed to test 40 patients with unipolar depression and a similar control group; final groups were of 8 depression patients and 5 control subjects, given aspartame 30 mg/kg/day or placebo for 7 days.	Patients with depression given aspartame experienced more frequent symptoms like nausea, depression, insomnia, temper, nervousness, dizziness, trouble remembering, fatigue, malaise, irritability.
Van den Eeden et al., 1994 [85]	Double-blind cross-over study on volunteers with self-identified headaches	18 subjects with headaches were randomly administered aspartame 30 mg/kg/day and placebo for 7 days in a 2-phase trial.	Headache incidence was 33% on aspartame regime vs. 24% on placebo (*p* = 0.04), with no significant difference in length of headache or occurrence of associated side effects.
Shaywitz et al., 1994 [87]	Randomized double-blind placebo-controlled crossover study	Unmedicated children with attention deficit disorders (DSM3 criteria) given aspartame 34 mg/kg/day single morning dose or placebo for 2 weeks were tested by parents at home and teacher (at school); during a 2-day admission to a study center cognitive tests and blood sampling were performed.	No changes in cognitive and behavioral tests (matching familiar figures, children’s checking task, airplane, Wisconsin card sorting, subjects’ treatment emergent symptom scale, multigrade inventory for teachers, Conners behavior rating scale) and biochemical values, except for plasma Phe and Tyr.
Stegink et al., 1995 [17]	Clinical trial including biochemistry and behavioral data on children described by their parents as sensitive to sugar vs. normal children	25 normal preschool children (aged 3–5) and 23 school-aged sugar-sensitive children (aged 6–10) were fed diets with high sucrose, aspartame, or saccharin for 3 weeks each, with blood samples at baseline (while fasting) and within the last 3 days of each dietary session.	No biochemical or behavioral abnormalities, except for a subject with low plasma α-Asp-Phe hydrolase activity (>2 SD below the mean) who experienced difficulty sitting still and paying attention towards the end of an aspartame diet session.
Spiers et al., 1998 [126]	Randomized double-blind placebo-controlled cross-over study	48 healthy volunteers kept in control conditions for 1 month, then fed with high (45 mg/kg/day), low (15 mg/kg/day) or no aspartame (placebo or sucrose) for 20 days; neuropsychology and laboratory testing was performed at 10 and 20 days under each regime.	Plasma phenylalanine concentrations increased significantly during aspartame diet. By sex and by therapy, amino acid, insulin, and glucose readings, EEG, unfavorable experiences, and neuropsychological tests results were compared: there were no significant changes.
Newman and Lipton 2001 [83]	Clinical case reports	Two recurrent migraine cases: 1. a 14-year-old male with a 2-year migraine history 2. a 36-year-old woman with migraines without an aura for 30 years.	After receiving treatment with a rizatriptan formulation that contains aspartame (Maxalt-MLT), both patients experienced headache aggravation.
Lindseth et al., 2014 [42]	Double-blinded repeated measures within-subjects neurobehavioral study to determine differences in cognition, mood, depression, headaches following consumption of high vs. low amounts of aspartame	28 healthy young adults (students) followed a study-prepared high-aspartame diet (25 mg/kg body weight/day) for 8 days and a low-aspartame diet (10 mg/kg body weight/day) for 8 days, with a 2-week washout period in between the diets.	Participants on high aspartame diets were more agitated (*p* = 0.002, paired *t* test), showed greater signs of depression (*p* = 0.001), and struggled on spatial orientation tests (*p* = 0.03), but with no effect on working memory.
Solomi et al., 2019 [127]	Cross-over design to test the acute glycemic effects of non-nutritive sweeteners aspartame and acesulfame-K	10 healthy volunteers (4 males, 6 females), with a mean age and BMI of 27.2 ± 6.9 years and 23.9 ± 2.4 kg/m^2^) were tested for glycemia while fasting and at 15-min intervals for 2 h after ingesting 236 mL of sucrose-sweetened cola with 125 mL of water, 25 g of glucose in 125 mL of water, and 25 g of glucose in 125 mL of water with 236 mL of diet cola.	None of the test beverages had significantly different glycemic responses; co-consuming artificially sweetened diet cola with a drink containing glucose had no discernible impact on postprandial glycaemia.
**IN VIVO STUDIES**			
***Neurological effects***			
Torii et al., 1986 [128]	Biochemistry and behavioral study in rats	Male Sprague–Dawley rats were fed with different combinations of casein, sucrose, corn starch, and aspartame (5% of diet weight) for 2 h (acute exposure) or for 3 weeks (chronic exposure).	Acute aspartame ingestion increased plasma and brain Phe and Tyr levels, but not Trp levels. Brain norepinephrine and dopamine levels were unaltered, serotonin levels were slightly increased on a protein-free diet. Chronic ingestion produced no significant chemical changes in brain.
Sharma and Coulombe 1987 [129]	Neurochemistry study in mice	Male CD-1 mice were given daily aspartame oral doses of 0, 13, 133, 650 mg/kg for 30 days.	Increases in adrenergic chemicals noticed after a single dose were not present upon chronic exposure. Serotonin was decreased in several brain regions, possibly due to the fact that increased Phe uptake decreased Trp uptake by brain tissue.
Goerss et al., 2000 [41]	Behavioral and neurochemistry study in rats	Aspartame (200–800 mg/kg *i*.*p*.) or a vehicle was given to adult male Long–Evans rats: 71 rats were used for behavioral testing, while 24 rats were used for a neurochemistry investigation.	High-dose aspartame dramatically reduced aggression, lengthened the intervals between attacks, reduced the number of bites per session, and markedly raised serotonin levels in the striatum.
Christian et al., 2004 [36]	Behavioral and neurochemistry study of chronic aspartame consumption in rats	Male Sprague–Dawley rats (225 g) that received aspartame (250 mg/kg/day) in water for three to four months vs. control rats. The animals were tested for latency to reward retrieval in a T-maze; after brain removal, membrane preparations from specific areas or whole brain were assessed for binding of [^3^H]quinuclidinyl benzilate and Na/K ATPase activity.	In aspartame-treated animals the number of muscarinic chlolinergic receptors increased by 31%; the frontal cortex, midcortex, posterior cortex, hippocampus, hypothalamus, and cerebellum all revealed substantial increases in muscarinic receptor densities, as well as significant increases in Na/K ATPase activity only in the midbrain. Latency to reward increased significantly in aspartame group after 3 and 4 months.
Collison et al., 2012 [39]	Behavioral and biochemistry study on spatial cognition, learning, memory, and insulin sensitivity in mice exposed to aspartame	C57Bl/6J mice of both sexes were exposed to aspartame or control diet since in utero until 17 weeks of age.	Male aspartame-fed mice gained weight, had higher fasting blood sugar levels (noticed also in females) and lower insulin sensitivity at 17 weeks compared to controls (*p* < 0.05). Male aspartame-fed mice had longer escape latencies during spatial learning trials in the Morris water maze test.
Iyashwamy et al., 2015 [38]	Neurobehavioral and biochemistry study in rats	Wistar male albino rats (200–220 g, control or kept on folate-deficient diet 45 days and methotrexate-treated for 1 week) were given aspartame 40 mg/kg/day or saline *p*.*o*. for 90 days. Anxiety was assessed by open field and elevated maze plus tests. Subsequently animals were sacrificed, blood (for formate levels) and brain samples (for hydrogen peroxide, immunohistochemistry, Western blot immunoassay and RT-PCR for TNF-a, JNK3, Fas, Caspase 8 and 9 vs. β-actin) were collected.	Aspartame and methotrexate-treated rats showed immobility, fecal bolus and a clearly diminished level of ambulation, rearing, and grooming, increased anxiety, increased plasma formate, increased hydrogen peroxide generation in different brain areas, increased expression, and neuronal staining with apoptosis markers.
Iyashwamy et al., 2018 [19]	Behavioral and neurobiochemistry study in rats	The following three groups of male Wistar albino rats were selected randomly: saline control, folate-deficient (MTX-treated) control, and folate-deficient (MTX-treated) group fed with aspartame 40 mg/kg for 3 months.	Aspartame-treated rats showed decline in memory tests (Morris water maze, Y maze), reduced body weight, increased plasma corticosterone levels, nitric oxide production, ACh-esterase activity, c-Fos, hsp70, iNOS and nNOS expression, microglia and astrocyte activation, as well as reduced Na/K ATPase activity and expression of NMDAR1, PSD95, synaptophysin, ERK, CaMKII, CREB in different brain regions.
Magalhães et al., 2019 [40]	Behavioral and electrophysiology study in rats	80 newborn Wistar rats were placed into 4 groups, each receiving aspartame 75 mg/kg/day or 125 mg/kg/day (groups ASP75 and ASP125), water (vehicle group), or no treatment (naïve group).	Early aspartame ingestion resulted in weight loss, anxiety (shorter times in open arms in elevated maze-plus test), and decreased cortical spreading depression (CSD) velocity via in vivo electrical brain recordings.
***Neurological effects of aspartate***			
Park et al., 2000 [118]	Behavioral and histopathology study in mice	Male ICR mice (6–7 weeks, 25–30 g) were administered a unique dose of 4.0 mg/g monosodium glutamate (MSG) or 0.5 mg/g aspartate (Asp) *i*.*p*., or the same amount of saline solution in the control groups.	MSG or Asp significantly damaged neurons in the arcuate nucleus of the hypothalamus, with no alterations in the cerebral cortex or hippocampus, or in any other brain regions. No significant changes were found in spontaneous motor activity, tail-flick response, but there was a decreased latency in passive avoidance test.
Vences-Mejía et al., 2006 [130]	Biochemistry study of liver and brain detoxifying enzymes in rat	24 male Wistar rats (21 days old) were given by gavage Asp 75 and 125 mg/kg body weight daily for 30 days. Liver, cerebrum, and cerebellum microsomes were assessed for activity of alkoxyresorufin O-dealkylase, 4-nitrophenol hydroxylase, erythromycin N–demethylase, and for different CytP450 isoforms by immunoblotting.	75 mg/kg Asp reliably increased the activity of all seven enzymes in the cerebrum and cerebellum, but not at the same levels.
Errico et al., 2008 [122]	Behavioral, biochemistry, histology, and electrophysiology study to assess the role of D-aspartate in regulating hippocampal synaptic plasticity	Male wild-type and mutant C57Bl/6 mice with targeted homozygous deletion of the D-aspartate oxidase (Ddo^−/−^) gene were fed with D-aspartate 20 mM *p*.*o*. for 1 month.	D-aspartate diet promoted long-term potentiation in hippocampal slices from both genetic and pharmacologically altered animal models, but it had no effect on the fundamental features of synaptic transmission; it slightly decreased spatial cognitive flexibility but not hippocampus-dependent learning and memory.
***Free radicals production/oxidative stress***			
Mourad and Noor 2011 [114]	Biochemistry study in rats	Adult male Wistar albino rats (120–180 g) were given aspartame 40 mg/day *p*.*o*. for 2, 4, or 6 weeks, with equivalent control groups. Brain homogenate samples were subjected to biochemical tests.	A significant decrease in lipid peroxidation occurred after 2 weeks of aspartame diet, followed by a significant increase after 4 weeks; reduced glutathione levels and increased superoxide dismutase activity were recorded after 4 and 6 weeks, while catalase activity increased after 6 weeks of aspartame diet.
Iyaswamy and Rathinasamy 2012 [34]	Neurobiochemistry study in rats	Male albino Wistar rats (200–220 g) were split into the following 3 groups: a saline control group, a methotrexate-treated control group, and a methotrexate-treated aspartame-fed group (75 mg/kg *p*.*o*. for 90 days on a folate-deficient diet).	Aspartame induced a significant increase in lipid peroxidation, superoxide dismutase, catalase, and glutathione peroxidase activity, decreased reduced glutathione and protein thiol levels, as well as detectable blood methanol levels.
Abdel-Salam et al., 2012 [31]	Neurobiochemistry study in mice	Male Swiss albino mice (20–22 g) were split into the following 10 groups: one saline control group (0.1 mL *i*.*p*.), 6 groups treated with lipopolysaccharide (LPS) 100 µg *i*.*p*. followed by aspartame 0, 0.625, 1.875, 5.625, 11.25, and 22.5 mg/kg *s*.*c*., and 3 groups treated with aspartame alone 11.25, 22.5, and 45 mg/kg *s*.*c*.	Aspartame after LPS decreased lipid peroxidation, reduced glutathione (GSH) and nitrite concentrations in brain and liver. Aspartame alone increased lipid peroxidation, TNF-a, and decreased GSH. Serotonin, noradrenaline, and dopamine in the brain were inhibited by aspartame in a dose-dependent manner. Nitrite, GSH, AST, ALT, ALP levels in the liver were not affected by aspartame. Oxidative stress and inflammation increased in the brain, but not in the liver.
Abhilash et al., 2013 [32]	Neurobiochemistry and histopathology study in rats	Three groups of male Wistar rats, (150–175 g) were created at random; the first group received aspartame 500 mg/kg in 3 mL water, the second group 1000 mg/kg, and the control group only 3 mL water daily for 180 days.	The group that received 1000 mg/kg featured decreased brain concentrations of reduced glutathione (GSH) and glutathione reductase activity; the group fed with 500 mg/kg showed only a significant reduction in GSH. Histopathological examination revealed mild vascular congestion in the 1000 mg/kg group.
Prokić et al., 2014 [20]	A study of oxidative status in erythrocytes of rats on aspartame diet	There were two groups of animals: the experimental group received aspartame 40 mg/kg *p*.*o*. daily for 6 weeks, whereas the control group received only water.	Superoxide anion, hydrogen peroxide, peroxynitrite, and lipid peroxides concentrations were significantly higher in the erythrocytes of the aspartame-treated group. Reduced glutathione (GSH) levels and catalase activity both increased under aspartame treatment.
Iyyaswamy and Rathinasamy 2014 [35]	Neurobiochemistry and histopathology study in rats	Adult male Wistar albino rats (200–220 g) were divided into the following three groups: saline control, folate-deficient (methotrexate-treated), and folate-deficient treated with aspartame 40 mg/kg *p*.*o*. for 90 days. Subsequently brain samples and homogenates were obtained.	Aspartame exposure led to significantly increased levels of protein carbonyl and decreased levels of protein thiol, increased lipid peroxidation, plasma methanol, and activity of superoxide dismutase, glutathione-S-transferase, glutathione peroxidase, catalase, significantly decreased levels of GSH, glutathione reductase. Proapoptotic gene expression was increased, as well as apoptosis markers caspase 3 and Bax, and CA1-3 pyramidal layer was depleted.
Choudhary and Rathinasamy 2014 [26]	Neurobehavioral and oxidative stress study in rats	Adult male Wistar albino rats (200–220 g) were fed a folate-deficient diet (FD) for 37 days and were given methotrexate (1 mg/kg) *i*.*v*. every second day for two weeks. The aspartame groups received aspartame 40 mg/kg/day *p*.*o*. 90 days.	After 90 days of aspartame administration, there was no discernible change in motor behavior, but there was a considerable decrease in membrane-bound ATPase activity and a decline in both enzymatic and non-enzymatic antioxidant levels in spinal cord lysates.
Alkafafy et al., 2015 [33]	Oxidative stress study on the rat liver	25 male Wistar albino rats aged 7 weeks were separated into 5 groups. The first 2 groups were given aspartame *p*.*o*. (250–1000 mg/kg), while groups 3–4 received saccharin (25 and 100 mg/kg, respectively), daily for 8 weeks.	The aspartame-treated groups showed increased liver enzymes activities, decreased antioxidant levels; all sweetener-treated groups showed histological hepatotoxic effects, downregulation of the tumor suppressor gene P27 and overexpression of the main oncogene H-Ras, pointing to a possible risk of liver carcinogenesis.
***Metabolic and other toxic effects***			
Abdel-Salam et al., 2009 [44]	A study on protective effects of citric acid and aspartame against CCl_4_-induced hepatic injury in rats	Adult Sprague–Dawley rats of both sexes (10 weeks, 120 g) were divided into the following eight groups: 1 control group and 7 groups treated with CCl_4_ in olive oil (1:1 *v*/*v*) 2.8 mg/kg; these groups also received silymarin (25 mg/kg), aspartame (0.625 or 1.25 mg/kg), or citric acid (10 mg/kg, 100 mg/kg, or 1000 mg/kg) *p*.*o*. daily for 1 week.	At 0.625 or 1.25 mg/kg, aspartame decreased plasma ALT, AST, and ALP, respectively, by 39.8–52.0%, 43.2–52.4%, and 50.0–68.5%. On histology, 1.25 mg/kg aspartame significantly decreased CCl_4_-induced vacuolar degeneration and necrosis.
Kim et al., 2011 [45]	Behavioral, biochemistry, and histopathology study on zebrafish	Zebrafish were given a high-cholesterol diet (HCD) along with aspartame or saccharin (5.2% and 3.6% of total food weight) for 12 days.	30% of the aspartame-HCD group died (vs. 0% in the saccharin and control groups). The aspartame-fed group showed acute swimming defects, a significant rise in blood glucose (up to 125 mg/dL), with more inflammatory cells in brain and liver.
Choudhary and Rathinasamy 2016 [46]	A study of expression of pro/antiapoptotic genes in immune organs of rats	Male Wistar albino rats (200–220 g) were fed a folate-deficient diet for 37 days, followed by methotrexate (MTX) every second day for two weeks; subsequently, they received aspartame 40 mg/kg/day (or saline in control) for 90 days.	Aspartame treatment did not result in obvious DNA fragmentation in the spleen, thymus, or lymph nodes; it also did not significantly alter the mRNA levels of Bcl-2 and Bax in the immune organs, while Hsp70 expression increased significantly.
Gul et al., 2017 [47]	A study of metabolic syndrome based on intestinal alkaline phosphatase inhibition by Phe	6-week-old male mice fed a high-fat diet (HFD) or normal diet received aspartame in water (0.96 mg/mL) or regular water for 18 weeks. In an acute in vivo model, an intestinal pouch was created by isolating a 6-cm segment of small bowel and was injected with aspartame 34 mg/kg or control saline. *In vitro* experiments monitored alkaline phosphatase (ALP) activity in the presence or absence of regular or aspartame diet soda.	Mice on HFD + aspartame gained more weight than the HFD + water group (48.1 vs. 42.4, *p* = 0.0001), and showed a higher glucose intolerance (*p* = 0.008); both HFD and normal diet + aspartame groups showed increased TNF-a levels. Aspartame lowered ALP activity in both in vivo and *in vitro* experiments (*p* = 0.02 and *p* = 0.034, respectively).
Helal et al., 2019 [131]	Biochemistry study in rats	30 male albino rats (100–120 g) were fed for 30 days with aspartame (50 mg/kg), acesulfame-K (15 mg/kg), or control diet.	Aspartame-treated rats had higher levels of serum glucose, insulin, creatinine, urea, lipid profiles (excepting HDL-C), higher ASAT and ALAT activities; serum testosterone, T3 and T4 levels decreased in the aspartame group, while total protein, albumin, and albumin/globulin ratio increased in both treated groups compared to control.
Nettleton et al., 2020 [48]	Study of an obesogenic diet effects on metabolism, gut microbiota, and mesolimbic reward system in rat dams and their offspring	Following obesity induction, 150 female Sprague–Dawley rats were divided into the following three groups: 1. high fat/sucrose diet (HFS) + water (obese-WTR); 2. HFS + aspartame 5–7 mg/kg/day (obese-APM); 3. HFS + stevia 2–3 mg/kg/day (obese-STV). Infants were weaned onto a control diet and given water, then monitored for 18 weeks.	Despite no direct low-calorie sweetener consumption by children, maternal low-calorie sweetener use together with HFS may affect weight management, glucose homeostasis, gut microbiota in dams and their offspring, particularly in early life. Mesolimbic reward pathway was altered in offspring of aspartame or stevia-fed dams as follows: increased ventral tegmental area dopamine transporter and tyrosine hydroxylase (only group 2) mRNA levels, increased nucleus accumbens D2 and µ opioid receptor levels.
Ragi et al., 2021 [7]	Biochemistry and metabolism study in rats	Adult male Sprague–-Dawley rats (7-week-old) were given a 1-week adaptation time, followed by aspartame (0.05% *w*/*w*) or sucralose (0.016% *w*/*w*) administration in diet, water, or both, for 7 weeks.	Aspartame consumption considerably increased body weight and fat mass, mostly because of an improvement in energy efficiency. Rather than the method of intake, the impact was correlated with the dosage. Additionally, use of aspartame was linked to glucose intolerance.
***Effects on immunity, inflammation, development***			
Pórtela et al., 2007 [28]	A morphometric and kariometric study in pregnant rats	20 pregnant rats, distributed randomly in 4 groups, received either aspartame 14 mg/kg or control in water at normal temperature or 40 °C; placentas, umbilical cords, and fetal livers (1000 hepatocytes) were analyzed morphometrically by kariometry.	The groups on aspartame at room temperature or 40 °C showed reduced placental, maternal, and fetal weight, umbilical cord length, as well as altered hepatocyte kariometric parameters.
Choudhary et al., 2014 [99]	Biochemistry, oxidative stress, and immune function study in rats	Adult male Wistar albino rats (12 weeks of age, 200–220 g), fed a folate-deficient diet for 37 days followed by methotrexate 1 mg/kg *i*.*v*. every second day for 2 weeks, were treated with aspartame 40 mg/kg *p*.*o*. for 15, 30, and 90 days, followed by venous blood collection.	In aspartame-fed groups there was a progressive decrease in RBC membrane-bound ATPase, G-6-PD and GR activity, increased lipid peroxidation and NO levels in RBC, neutrophils, and lymphocytes, decreased neutrophil adhesion and phagocytic index, increased antibody titers and soluble immune complexes.
Shalaby et al., 2019 [29]	Histological/ immunohistochemical study of the placenta in rats	20 pregnant female rats received aspartame 14 mg/kg *p*.*o*. or control during days 9–11 of pregnancy.	Aspartame treatment decreased placental weight and thickness of labyrinth and basal zones, induced rupture of interhemal membranes, lysis of trophoblast cells, as well as increased VEGF staining of labyrinth and basal zones.
Fareed and Mostafa 2021 [49]	Biochemical and histological study on renal maturation in rat offspring	Pregnant rats were randomly divided into the following four groups: 1. control group; 2. aspartame group: 40 mg/kg/day until postnatal day 30; 3. caffeine group: 80 mg/kg/day; 4. aspartame and caffeine group.	Group 4 showed in kidneys a substantial increase in oxidative load (malondialdehyde), reduced antioxidant enzymes and total glutathione activity (superoxide dismutase and glutathione peroxidase). Renal tissues in group 4 matured faster than in groups 2 and 3, but with more pathological changes.
***Genotoxic effects, mutagenic and carcinogenic potential***			
Bandyopadhyay et al., 2008 [50]	Genotoxicity study by comet assay in the bone marrow cells of mice	Swiss albino mice (8–10 weeks, 25 g) were orally administered aspartame (7, 14, 28, 35 mg/kg), acesulfame-K (150, 300, 600 mg/kg), and saccharin (50, 100, 200 mg/kg) individually. The animals were sacrificed after 18 h and the bone marrow cells were processed for comet assay.	As evidenced by increased comet-tail extent and % DNA in the tail, sweetener-induced DNA strand breaks enhanced the comet characteristics of DNA in the bone marrow cells. Acesulfame-K and saccharin caused more DNA damage than aspartame.
Landrigan and Straif 2021 [8]	Immunohistochemical and morphological reevaluation of a study performed at Ramazzini Institute in 2006–2007 on rats and mice with aspartame-induced tumors	Reevaluation of studies BT6008 (aspartame 0–100,000 ppm since week 8 in rats), BT6009 (prenatal exposure in rats), BT6010 (prenatal exposure in Swiss mice)—a total of 2270 Sprague–Dawley rats and 852 Swiss mice.	In 92.3% of cases, the immunohistochemical and morphological reevaluation supported the initial diagnosis of malignancy; 3 lesions were reclassified as lymphoid hyperplasia and 3 as chronic inflammation with fibrosis out of the 6 lesions originally identified as lymphomas (8% of all HLTs). No signs of *Mycoplasma* infection were seen.
***IN VITRO* STUDIES**			
***Neurological effects***			
Fountain et al., 1988 [51]	*In vitro* study of hippocampal slice excitability and long-term potentiation (LTP)	Female Long–Evans rats aged 60–90 days used to prepare 400–450 µm thick hippocampal slices.	Exposure to 0.01, 0.1, 1, and 10 mM aspartame potentiated the electrical response of CA1 neurons (increased fEPSP slope and amplitude), similar to 0.1 mM aspartate, phenylalanine or its methyl ester, without effects on inhibitory systems (tested by double-pulse protocols) and LTP.
Pan-Hou et al., 1990 [30]	Ligand-receptor binding assay	Binding of NMDA, aspartate, and aspartame in competition with ^3^H-glutamate on NMDARs from rat brain synaptic membrane preparations.	0.1 mM NMDA, 1 mM aspartate, and 1 mM aspartame displaced >50% of ^3^H-glutamate without modifying *V*_max_ of glutamate binding, suggesting competitive binding.
Simintzi et al., 2007a [52]	Acetylcholine esterase activity in rat hippocampus and that of pure enzyme	Rat hippocampus homogenates or pure AChE enzyme were incubated with aspartame metabolites (aspartate 0.82–10 mM, phenylalanine 0.07–0.5 mM, methanol 0–0.8 mM).	Reduced AChE activity at high doses of metabolites (equivalent to consumption of aspartame 150–200 mg/kg).
Simintzi et al., 2007b [37]	Na/K ATPase activity in rat hippocampus and that of pure enzyme	Rat hippocampus homogenates or pure Na/K ATPase were incubated with aspartame metabolites (aspartate 0.82–10 mM, phenylalanine 0.07–0.5 mM, methanol 0–0.8 mM)	Reduced Na/K ATPase activity in homogenates (but increased activity of pure enzyme) at higher doses of metabolites (equivalent to consumption of aspartame 34–200 mg/kg).
***Cytotoxic, genotoxic, and carcinogenic effects***			
Kashanian et al., 2013 [53]	DNA binding study	Native calf thymus DNA interaction with aspartame at physiological pH was studied by spectrophotometry, spectrofluorimetric competition, and circular dichroism (CD); aspartame fluorescence quenching by DNA at various temperatures was used to estimate the number of binding sites per base pair.	The UV absorption band of aspartame exhibits hypochromism and red shift. Fluorescence quenching by DNA provided binding constants and corresponding number of binding sites; enthalpy (+181 kJ/mol) and entropy (+681 J/mol·K) changes were estimated. Iodide, methylene blue, and competitive Hoechst 22358-aspartame quenching experiments proved minor groove DNA binding of aspartame, and lack of CD spectra changes with aspartame indicate a non-intercalative interaction.
Pandurangan et al., 2015 [27]	Cytotoxicity study on human cervical carcinoma cells	HeLa cells were seeded at a density of 2.2 × 10^5^ cells/well in 6-well plates. After 24 h, the cells were treated with aspartame at different concentrations (10 µM, 100 µM, 1 mM, 10 mM, 20 mM) for 24 and 48 h.	After exposure to greater aspartame concentrations, cell viability was considerably changed. At greater aspartame exposure doses, ROS produced by mitochondria increased. DNA fragmentation occurred upon exposure to aspartame 10 or 20 mM. At 1–20 mM aspartame concentrations, apoptotic and necrotic bodies were discovered.
Park et al., 2019 [117]	Cytotoxicity study on embryonic mouse hypothalamic cell line mHypoE-N43/5	mHypoE-N43/5 cells were cultured in medium containing either vehicle (saline) or 0.5, 5, 10, 20 mM of sucralose, aspartame, acesulfame-K, or rebaudioside A (all from Sigma, St. Louis, Missouri, USA) for 48 h. All non-nutritive sweetener stock solutions were made in sterile water.	When given in tolerable amounts for daily consumption, rebaudioside A did not cause ER stress, but sucralose, aspartame, and acesulfame-K did. Arcuate nucleus explants axon outgrowth was unaffected by sucralose, aspartame, or rebaudioside A, and aspartame had no impact on caspase 3/7 activity.
Maghiari et al., 2020 [22]	Cytotoxicity study on HT-29 human colorectal carcinoma cells and chicken egg embryos	HT-29 human colorectal carcinoma cells were cultured in specific medium, and incubated with different concentrations of aspartame (0.1, 0.25, 0.5, 1, 3, 6, 15, 30, or 50 mM) for 72 h, followed by Alamar blue cell viability assay. Chorioallantoic membranes of eggs were exposed to 10 µL aspartame solution placed inside a ring.	At the highest aspartame doses examined (15, 30, and 50 mM), there was a dose-dependent cytotoxic effect with a considerable reduction in viable cells, as well as morphological cellular alterations. Aspartame (15 and 30 mM) was shown to have a pro-angiogenic effect *in ovo* as well as a negligible irritating potential.
Çadirci et al., 2020 [54]	An *in vitro* cytotoxicity, genotoxicity, and oxidative stress study on cultured human blood cells	Cytotoxicity was studied via MTT and lactate dehydrogenase release tests, genotoxic damage potential by using chromosome aberration (CA) assay, and antioxidant/oxidant activity by using total antioxidant capacity (TAC) and total oxidative stress (TOS) analysis in cultured primary human whole blood cells.	Substantial, clearly concentration-dependent declines in cell viability were obtained upon aspartame exposure (3.125–100 mg/L). In aspartame-treated cells there was an increase in the frequency of CA, while TAC and TOS levels in whole blood cultures were not significantly altered.
Griebsch et al., 2023 [100]	Oxidative stress, membrane composition, and mitochondrial damage study	The SH-SY5Y human neuroblastoma cell line was exposed to aspartame (271.7 μM) or its metabolites (Asp, Phe, methanol).	Aspartame and metabolites treatment altered mitochondrial integrity (assessed by transmission electron microscopy), increased total mitochondrial and lipid droplets area, decreased cardiolipin levels to 56.7 ± 5.6% (*p* = 0.011), activated mitophagy and ROS release, increased expression of FIS1, PINK1, SOD1 and 2, increased triacylglycerol, phosphatidylcholine, and phosphatidylethanolamine species.
***Angiogenesis effects***			
Alleva et al., 2011 [23]	*In vitro* angiogenesis and cytotoxicity study	Human endothelial cells were co-cultured with fibroblasts in a standardized angiogenesis model (Angio-Kit). Human umbilical vein endothelial cells (HUVEC) and fibroblasts were cultured *in vitro*. All cultures were exposed to aspartame 20, 40, 60, 80, or 100 µM dissolved in complete culture medium.	Exposure to aspartame stimulated angiogenesis, and also ROS production in endothelial cells associated with cytotoxicity (increased Erk1/2 and p38 activation and IL-6 secretion), but not in IMR-90 fibroblasts.
Yesildal et al., 2015 [25]	*In vitro* and *in vivo* angiogenesis and wound healing study	Male Sprague–Dawley rats (8 weeks, 200–250 g) were used to remove two circular slices of skin (5 mm diameter). The wounds were treated with PBS or aspartame 50 mM for 7 days and collected on day 8, after calculating the surface area. Chicken eggs choriallantoic membrane (CAM) angiogenesis was tested by application of aspartame 6–60 mM, and HUVEC cells exposed to aspartame 20–100 µM were used for tube formation and 2,3-bis-2H-tetrazolium-5-carboxanilide (XTT) viability assays.	Aspartame increased CAM angiogenesis in a dose-dependent manner (*p* < 0.001) and improved wound healing (*p* < 0.05). Aspartame also slightly increased HUVEC cell proliferation (not statistically significant) and had no effect on tube formation.
Enuwosa et al., 2021 [24]	*In vitro* study of effects of artificial sweeteners on VEGF-induced permeability of glomerular endothelium	Human primary glomerular microvascular endothelial cells (GMVEC) in culture were treated with increasing concentrations (0.1–100 µM) of aspartame, saccharin, and sucralose for 24 h, and tested for endothelial monolayer permeability with dextran 20 kDa-FITC upon VEGF exposure, VE-cadherin expression and intracellular cAMP level by ELISA, ROS production, and GC-MS for sucralose concentration in cell lysates.	All tested sweeteners had no effect on traditional VEGF signaling, but only saccharin and sucralose protected against VEGF-induced permeability, dependent on the sweet taste receptor T1R3. VEGF induced an increase in ROS production, which was not influenced by any sweetener. All sweeteners maintained VE-cadherin expression during VEGF exposure. In the absence of VEGF, aspartame significantly increased oxidative stress in GMVEC, while sucralose and saccharin had no effect.
***Other effects***			
Manion et al., 2001 [55]	*In vitro* and clinical study of the efficacy of aspartame in treating sickle cell anemia	Twenty children with sickle cell anemia provided heparinized blood samples exposed to aspartame 1 or 2 mg/mL. 23 other patients with homozygous/heterozygous HbS or HbS/β^0^ were given aspartame 1.5, 3, or 6 mg/kg single-dose, and their blood samples were monitored up to 1440 min afterwards.	1 mg/mL reduced sickled cells from 28% to 14%, and even further with 2 mg/mL. In 15 individuals with homozygous HbS anemia, sickling was prevented by 6 mg/kg aspartame for at least 6 h.
Rios et al., 2018 [56]	Comparison of enamel erosion produced by regular and light colas, with addition of aspartame sweetener	60 bovine enamel blocks were exposed 2 min. 4 times daily for 5 consecutive days to erosion in 5 varieties of cola and kept in artificial saliva between exposures. Experimental groups: RC-regular cola-degassed (pH 2.6), RCpH-a base was added to raise the pH of RC (pH 3.0), RCAS aspartame added to RC (pH 2.6), LC-light cola (pH 3.0), LCpH-acid added to LC (pH 2.6).	The % surface hardness change in enamel after 1 day was similar for all 5 groups. LC caused less enamel loss than RC, but differences were not significant (*p* > 0.05) between erosion and erosion plus abrasion for LC. However, for RC erosion plus abrasion resulted in higher enamel loss than erosion alone. LCpH had an erosion effect similar to RC, while RCpH had similar effect to LC.
Pandurangan et al., 2014 [57]	An *in vitro* study of aspartame effects on preadipocyte differentiation	3T3-L1 mouse preadipocytes were cultured and differentiated for 6 days in the absence and presence of aspartame 10 μg/mL.	The induction of p-PPARγ, PPARγ, SREBP1, and adipsin (by Western blot) and PPARγ, FABP4, and C/EBPα (RT-qPCR) was significantly reduced in aspartame-treated preadipocytes, as well as lipid accumulation (by Oil Red O staining).
Sun et al., 2019 [58]	*In vivo* and *in vitro* study on small intestinal cell cycle and stimulating secretion and expression of glucagon-like peptide-2 (GLP-2) in pre-weaned lambs	Twelve 14-day lambs were randomly divided into two groups; control (*n* = 6) fed on starter food, and aspartame-fed (200 mg/kg) (*n* = 6) up to 49 days. At 56 days, 4 healthy lambs’ jejunal tissue was used to dissociate and culture epithelial cells, treated with control, GLP-2 10 nM alone or with IGF-1R inhibitor picropodophyllin (PPP) 1 µM.	Aspartame-fed lambs showed higher GLP-2 plasma concentrations (*p* < 0.05), and larger jejunum weight/live body weight and jejunal crypt depth, as well as increased expression of cyclins D1, A2, CDK4 and 6, glucagon, IGF-1, GLP-2R in jejunum/ileum. Jejunal cells treated with GLP-2 (2h) showed increased proliferation (MTT test) and expression of IGF-1, cyclin D1, CDK6, which were decreased by PPP.
Chontzopoulou et al., 2021 [59]	*In silico* and experimental study on lipoxygenase (LOX) inhibition	The following different LOX isoforms crystal structures were used for in silico studies: docking, molecular dynamics (MD) followed by QM/MM geometry optimization of ligand-receptor complexes. For *in vitro* assays of saturation transfer difference NMR (STD-NMR), a stock solution (10 mM) of the tested compound was prepared in DMSO.	*In silico* and *in vitro* assays confirmed strong aspartame binding to LOX-1 isoform (IC50 = 50 ± 3.0 μΜ) with functional inhibition. These results suggest that aspartame could serve as a novel starting point for drug design of LOX inhibitors.

## References

[1] Chattopadhyay S., Raychaudhuri U., Chakraborty R. (2014). Artificial sweeteners—A review. J. Food Sci. Technol..

[2] Marinovich M., Galli C.L., Bosetti C., Gallus S., La Vecchia C. (2013). Aspartame, low-calorie sweeteners and disease: Regulatory safety and epidemiological issues. Food Chem. Toxicol..

[3] Mazur R.H., Stegink L.D., Filer L.J.J. (1984). Discovery of aspartame. Aspartame: Physiology and Biochemistry.

[4] Mazur R.H., Schlatter J.M., Goldkamp A.H. (1969). Structure-taste relationships of some dipeptides. J. Am. Chem. Soc..

[5] Tobey N.A., Heizer W.D. (1986). Intestinal hydrolysis of aspartylphenylalanine—The metabolic product of aspartame. Gastroenterology.

[6] Barceloux D.G., Bond G.R., Krenzelok E.P., Cooper H., Vale J.A. (2002). American Academy of Clinical Toxicology practice guidelines on the treatment of methanol poisoning. J. Toxicol. Clin. Toxicol..

[7] Ragi M.E., El-Haber R., El-Masri F., Obeid O.A. (2021). The effect of aspartame and sucralose intake on body weight measures and blood metabolites: Role of their form (solid and/or liquid) of ingestion. Br. J. Nutr..

[8] Landrigan P.J., Straif K. (2021). Aspartame and cancer—New evidence for causation. Environ. Health.

[9] Stegink L.D., Brummel M.C., Filer L.J., Baker G.L. (1983). Blood methanol concentrations in one-year-old infants administered graded doses of aspartame. J. Nutr..

[10] Stegink L.D., Brummel M.C., McMartin K., Martin-Amat G., Filer L.J., Baker G.L., Tephly T.R. (1981). Blood methanol concentrations in normal adult subjects administered abuse doses of aspartame. J. Toxicol. Environ. Health.

[11] Stegink L.D., Filer L., Tschanz C., Butchko H.H., Stargel W., Kotsonis F.N. (1996). Effects of Aspartame Ingestion on Plasma Aspartate, Phenylalanine and Methanol Concentrations in Potentially Sensitive Populations. The Clinical Evaluation of a Food Additive: Assessment of Aspartame.

[12] Stegink L.D., Filer L.J., Baker G.L. (1987). Plasma amino acid concentrations in normal adults ingesting aspartame and monosodium L-glutamate as part of a soup/beverage meal. Metabolism.

[13] Stegink L.D., Filer L.J., Baker G.L. (1988). Repeated ingestion of aspartame-sweetened beverage: Effect on plasma amino acid concentrations in normal adults. Metabolism.

[14] Stegink L.D., Filer L.J., Baker G.L., Brummel M.C. (1979). Plasma and erythrocyte amino acid levels of adult humans given 100 mg/kg body weight aspartame. Toxicology.

[15] Stegink L.D., Filer L.J., Baker G.L., McDonnell J.E. (1980). Effect of an abuse dose of aspartame upon plasma and erythrocyte levels of amino acids in phenylketonuric heterozygous and normal adults. J. Nutr..

[16] Stegink L.D., Filer L.J., Bell E.F., Ziegler E.E., Tephly T.R. (1989). Effect of repeated ingestion of aspartame-sweetened beverage on plasma amino acid, blood methanol, and blood formate concentrations in normal adults. Metabolism.

[17] Stegink L.D., Lindgren S.D., Brummel M.C., Stumbo P.J., Wolraich M.L. (1995). Erythrocyte L-aspartyl-L-phenylalanine hydrolase activity and plasma phenylalanine and aspartate concentrations in children consuming diets high in aspartame. Am. J. Clin. Nutr..

[18] Walton R.G., Hudak R., Green-Waite R.J. (1993). Adverse reactions to aspartame: Double-blind challenge in patients from a vulnerable population. Biol. Psychiatry.

[19] Iyaswamy A., Kammella A.K., Thavasimuthu C., Wankupar W., Dapkupar W., Shanmugam S., Rajan R., Rathinasamy S. (2018). Oxidative stress evoked damages leading to attenuated memory and inhibition of NMDAR-CaMKII-ERK/CREB signalling on consumption of aspartame in rat model. J. Food Drug Anal..

[20] Prokić M.D., Paunović M.G., Matić M.M., Djordjević N.Z., Ognjanović B.I., Štajn A.S., Saičić Z.S. (2014). Prooxidative effects of aspartame on antioxidant defense status in erythrocytes of rats. J. Biosci..

[21] Schernhammer E.S., Bertrand K.A., Birmann B.M., Sampson L., Willett W.C., Feskanich D. (2012). Consumption of artificial sweetener- and sugar-containing soda and risk of lymphoma and leukemia in men and women. Am. J. Clin. Nutr..

[22] Maghiari A.L., Coricovac D., Pinzaru I.A., Macașoi I.G., Marcovici I., Simu S., Navolan D., Dehelean C. (2020). High Concentrations of Aspartame Induce Pro-Angiogenic Effects in Ovo and Cytotoxic Effects in HT-29 Human Colorectal Carcinoma Cells. Nutrients.

[23] Alleva R., Borghi B., Santarelli L., Strafella E., Carbonari D., Bracci M., Tomasetti M. (2011). *In vitro* effect of aspartame in angiogenesis induction. Toxicol. Vitr..

[24] Enuwosa E., Gautam L., King L., Chichger H. (2021). Saccharin and sucralose protect the glomerular microvasculature *in vitro* against VEGF-induced permeability. Nutrients.

[25] Yesildal F., Aydin F.N., Deveci S., Tekin S., Aydin I., Mammadov R., Fermanli O., Avcu F., Acikel C.H., Ozgurtas T. (2015). Aspartame induces angiogenesis *in vitro* and in vivo models. Hum. Exp. Toxicol..

[26] Choudhary A.K., Rathinasamy S. (2014). Effect of aspartame in spinal cord and motor behavior in Wistar albino rats. J. Behav. Health.

[27] Pandurangan M., Enkhtaivan G., Mistry B., Chandrasekaran M., Noorzai R., Kim D.H. (2016). Investigation of role of aspartame on apoptosis process in HeLa cells -->. Saudi J. Biol. Sci..

[28] Pórtela G.S., Azoubel R., Batigália F. (2007). Effects of aspartame on maternal-fetal and placental weights, length of umbilical cord and fetal liver: A kariometric experimental study. Int. J. Morphol..

[29] Shalaby A.M., Ibrahim M., Aboregela A.M. (2019). Effect of aspartame on the placenta of adult albino rat. A histological and immunohistochemical study. Ann. Anat..

[30] Pan-Hou H., Suda Y., Ohe Y., Sumi M., Yoshioka M. (1990). Effect of aspartame on N-methyl-D-aspartate-sensitive L-[3H] glutamate binding sites in rat brain synaptic membranes. Brain Res..

[31] Abdel-Salam O.M., Salem N.A., Hussein J.S. (2012). Effect of aspartame on oxidative stress and monoamine neurotransmitter levels in lipopolysaccharide-treated mice. Neurotox. Res..

[32] Abhilash M., Sauganth Paul M., Varghese M.V., Nair R.H. (2013). Long-term consumption of aspartame and brain antioxidant defense status. Drug Chem. Toxicol..

[33] Alkafafy M.E.-S., Ibrahim Z.S., Ahmed M.M., El-Shazly S.A. (2015). Impact of aspartame and saccharin on the rat liver: Biochemical, molecular, and histological approach. Int. J. Immunopathol. Pharmacol..

[34] Iyaswamy A., Rathinasamy S. (2012). Effect of chronic exposure to aspartame on oxidative stress in the brain of albino rats. J. Biosci..

[35] Iyaswamy A., Rathinasamy S. (2014). Biochemical responses and mitochondrial mediated activation of apoptosis on long-term effect of aspartame in rat brain. Redox Biol..

[36] Christian B., McConnaughey K., Bethea E., Brantley S., Coffey A., Hammond L., Harrell S., Metcalf K., Muehlenbein D., Spruill W. (2004). Chronic aspartame affects T-maze performance, brain cholinergic receptors and Na+, K+-ATPase in rats. Pharmacol. Biochem. Behav..

[37] Simintzi I., Schulpis K.H., Angelogianni P., Liapi C., Tsakiris S. (2007). L-Cysteine and glutathione restore the reduction of rat hippocampal Na+, K+-ATPase activity induced by aspartame metabolites. Toxicology.

[38] Iyaswamy A., Rathinasamy S. (2015). Neurobehavioral changes and activation of neurodegenerative apoptosis on long-term consumption of aspartame in the rat brain. J. Nutr. Intermed. Metabol..

[39] Collison K.S., Makhoul N.J., Zaidi M.Z., Saleh S.M., Andres B., Inglis A., Al-Rabiah R., Al-Mohanna F.A. (2012). Gender dimorphism in aspartame-induced impairment of spatial cognition and insulin sensitivity. PLoS ONE.

[40] Magalhães P.C.G., Abadie-Guedes R., da Costa Mendonça M.A.B., de Souza A.D., Guedes R.C.A. (2019). Behavioral and electrophysiological brain effects of aspartame on well-nourished and malnourished rats. Metab. Brain Dis..

[41] Goerss A.L., Wagner G.C., Hill W.L. (2000). Acute effects of aspartame on aggression and neurochemistry of rats. Life Sci..

[42] Lindseth G.N., Coolahan S.E., Petros T.V., Lindseth P.D. (2014). Neurobehavioral effects of aspartame consumption. Res. Nurs. Health.

[43] Lim U., Subar A.F., Mouw T., Hartge P., Morton L.M., Stolzenberg-Solomon R., Campbell D., Hollenbeck A.R., Schatzkin A. (2006). Consumption of aspartame-containing beverages and incidence of hematopoietic and brain malignancies. Cancer Epidemiol. Biomark. Prev..

[44] Abdel Salam O.M., Shaffie N.M., Sleem A.A. (2009). Hepatoprotective effects of citric acid and aspartame on carbon tetrachloride-induced hepatic damage in rats. EXCLI J..

[45] Kim J.Y., Seo J., Cho K.H. (2011). Aspartame-fed zebrafish exhibit acute deaths with swimming defects and saccharin-fed zebrafish have elevation of cholesteryl ester transfer protein activity in hypercholesterolemia. Food Chem. Toxicol..

[46] Choudhary A.K., Rathinasamy S. (2016). Effects of aspartame on hsp70, bcl-2 and bax expression in immune organs of Wistar albino rats. J. Biomed. Res..

[47] Gul S.S., Hamilton A.R., Munoz A.R., Phupitakphol T., Liu W., Hyoju S.K., Economopoulos K.P., Morrison S., Hu D., Zhang W. (2017). Inhibition of the gut enzyme intestinal alkaline phosphatase may explain how aspartame promotes glucose intolerance and obesity in mice. Appl. Physiol. Nutr. Metab..

[48] Nettleton J.E., Cho N.A., Klancic T., Nicolucci A.C., Shearer J., Borgland S.L., Johnston L.A., Ramay H.R., Noye Tuplin E., Chleilat F. (2020). Maternal low-dose aspartame and stevia consumption with an obesogenic diet alters metabolism, gut microbiota and mesolimbic reward system in rat dams and their offspring. Gut.

[49] Fareed S.A., Mostafa H.E. (2021). Could aspartame exacerbate caffeine effects on renal maturation in rat’s offspring? A biochemical and histological study. Birth Defects Res..

[50] Bandyopadhyay A., Ghoshal S., Mukherjee A. (2008). Genotoxicity testing of low-calorie sweeteners: Aspartame, acesulfame-K, and saccharin. Drug Chem. Toxicol..

[51] Fountain S.B., Hennes S.K., Teyler T.J. (1988). Aspartame exposure and *in vitro* hippocampal slice excitability and plasticity. Fundam. Appl. Toxicol..

[52] Simintzi I., Schulpis K.H., Angelogianni P., Liapi C., Tsakiris S. (2007). The effect of aspartame on acetylcholinesterase activity in hippocampal homogenates of suckling rats. Pharmacol. Res..

[53] Kashanian S., Khodaei M.M., Kheirdoosh F. (2013). *In vitro* DNA binding studies of Aspartame, an artificial sweetener. J. Photochem. Photobiol. B.

[54] Çadirci K., Tozlu Ö.Ö., Türkez H., Mardinoğlu A. (2020). The *in vitro* cytotoxic, genotoxic, and oxidative damage potentials of the oral artificialsweetener aspartame on cultured human blood cells. Turk. J. Med. Sci..

[55] Manion C.V., Howard J., Ogle B., Parkhurst J., Edmundson A. (2001). Aspartame effect in sickle cell anemia. Clin. Pharmacol. Ther..

[56] Rios D., Ionta F.Q., Rebelato R., Jordão M.C., Wang L., Magalhães A.C., Honório H.M. (2018). The effect of aspartame and pH changes on the erosive potential of cola drinks in bovine enamel: An *in vitro* study. J. Clin. Exp. Dent..

[57] Pandurangan M., Park J., Kim E. (2014). Aspartame downregulates 3T3-L1 differentiation. Vitr. Cell. Dev. Biol. Anim..

[58] Sun D., Liu L., Mao S., Zhu W., Liu J. (2019). Aspartame supplementation in starter accelerates small intestinal epithelial cell cycle and stimulates secretion of glucagon-like peptide-2 in pre-weaned lambs. J. Anim. Physiol. Anim. Nutr..

[59] Chontzopoulou E., Papaemmanouil C.D., Chatziathanasiadou M.V., Kolokouris D., Kiriakidi S., Konstantinidi A., Gerogianni I., Tselios T., Kostakis I.K., Chrysina E.D. (2022). Molecular investigation of artificial and natural sweeteners as potential anti-inflammatory agents. J. Biomol. Struct. Dyn..

[60] (2013). EFSA Panel on Food Additives Nutrient Sources added to Food. Scientific Opinion on the re-evaluation of aspartame (E 951) as a food additive. EFSA J..

[61] Soffritti M., Belpoggi F., Degli Esposti D., Lambertini L., Tibaldi E., Rigano A. (2006). First experimental demonstration of the multipotential carcinogenic effects of aspartame administered in the feed to Sprague-Dawley rats. Environ. Health Perspect..

[62] Soffritti M., Belpoggi F., Tibaldi E., Esposti D.D., Lauriola M. (2007). Life-span exposure to low doses of aspartame beginning during prenatal life increases cancer effects in rats. Environ. Health Perspect..

[63] Collison K.S., Inglis A., Shibin S., Andres B., Ubungen R., Thiam J., Mata P., Al-Mohanna F.A. (2016). Differential effects of early-life NMDA receptor antagonism on aspartame-impaired insulin tolerance and behavior. Physiol. Behav..

[64] Sykes M. (2015). The Aspartame Controversy of 1981, The Hidden Truth Behind the Not-So-Sweet Artificial Sweetner. Va. Tech Undergrad. Hist. Rev..

[65] Butchko H., Stargel W., Comer C., Mayhew D., Benninger C., Blackburn G., De Sonneville L., Geha R., Hertelendy Z., Kostner A. (2002). Intake of aspartame vs the acceptable daily intake. Regul. Toxicol. Pharmacol..

[66] Fatibello-Filho O., Marcolino-Junior L.H., Pereira A.V. (1999). Solid-phase reactor with copper (II) phosphate for flow-injection spectrophotometric determination of aspartame in tabletop sweeteners. Anal. Chim. Acta.

[67] Fitch C., Keim K. (2012). Position of the academy of nutrition and dietetics: Use of nutritive and nonnutritive sweetener. J. Acad. Nutr. Diet..

[68] Monte W.C. (1984). Aspartame: Methanol and the public health. J. Appl. Nutr..

[69] Magnuson B.A., Burdock G.A., Doull J., Kroes R.M., Marsh G.M., Pariza M.W., Spencer P.S., Waddell W.J., Walker R., Williams G.M. (2007). Aspartame: A safety evaluation based on current use levels, regulations, and toxicological and epidemiological studies. Crit. Rev. Toxicol..

[70] National Research Council (1993). Pesticides in the Diets of Infants and Children.

[71] Olney J.W., Farber N.B., Spitznagel E., Robins L.N. (1996). Increasing brain tumor rates: Is there a link to aspartame?. J. Neuropathol. Exp. Neurol..

[72] Czarnecka K., Pilarz A., Rogut A., Maj P., Szymańska J., Olejnik Ł., Szymański P. (2021). Aspartame-True or False? Narrative Review of Safety Analysis of General Use in Products. Nutrients.

[73] Amchra F.Z., Al Faiz C., Chaouqi S., Khiraoui A., Benhmimou A., Guedira T. (2018). Effect of Stevia rebaudiana, sucrose and aspartame on human health: A comprehensive review. J. Med. Plants Stud..

[74] Singh M., Kumar A., Tarannum N. (2013). Water-compatible ‘aspartame’-imprinted polymer grafted on silica surface for selective recognition in aqueous solution. Anal. Bioanal. Chem..

[75] Bell L.N., Labuza T.P. (1991). Aspartame degradation kinetics as affected by pH in intermediate and low moisture food systems. J. Food Sci..

[76] Lipton W.E., Li Y.N., Younoszai M.K., Stegink L.D. (1991). Intestinal absorption of aspartame decomposition products in adult rats. Metabolism.

[77] Magnuson B.A., Carakostas M.C., Moore N.H., Poulos S.P., Renwick A.G. (2016). Biological fate of low-calorie sweeteners. Nutr. Rev..

[78] Hooper N.M., Hesp R.J., Tieku S. (1994). Metabolism of aspartame by human and pig intestinal microvillar peptidases. Biochem. J..

[79] Li D., Zhao X.H., Yang T.B., Johnson E.W., Thacker P.A. (1999). A comparison of the intestinal absorption of amino acids in piglets when provided in free form or as a dipeptide. Asian-Australas. J. Anim. Sci..

[80] Adibi S.A. (1971). Intestinal transport of dipeptides in man: Relative importance of hydrolysis and intact absorption. J. Clin. Investig..

[81] Stegink L.D. (1987). The aspartame story: A model for the clinical testing of a food additive. Am. J. Clin. Nutr..

[82] Koehler S.M., Glaros A. (1988). The effect of aspartame on migraine headache. Headache.

[83] Newman L.C., Lipton R.B. (2001). Migraine MLT-down: An unusual presentation of migraine in patients with aspartame-triggered headaches. Headache.

[84] Schiffman S.S., Buckley C.E., Sampson H.A., Massey E.W., Baraniuk J.N., Follett J.V., Warwick Z.S. (1987). Aspartame and susceptibility to headache. N. Engl. J. Med..

[85] Van den Eeden S.K., Koepsell T.D., Longstreth W.T., van Belle G., Daling J.R., McKnight B. (1994). Aspartame ingestion and headaches: A randomized crossover trial. Neurology.

[86] Ranney R.E., Oppermann J.A. (1979). A review of the metabolism of the aspartyl moiety of aspartame in experimental animals and man. J. Environ. Pathol. Toxicol..

[87] Shaywitz B.A., Sullivan C.M., Anderson G.M., Gillespie S.M., Sullivan B., Shaywitz S.E. (1994). Aspartame, behavior, and cognitive function in children with attention deficit disorder. Pediatrics.

[88] Choudhary A.K., Lee Y.Y. (2018). The debate over neurotransmitter interaction in aspartame usage. J. Clin. Neurosci..

[89] Humphries P., Pretorius E., Naudé H. (2008). Direct and indirect cellular effects of aspartame on the brain. Eur. J. Clin. Nutr..

[90] Reinhard J.F., Reinhard J.F.J., Vida J.A. (1972). Experimental evaluation of anticonvulsants. Anticonvulsants.

[91] Maher T.J., Wurtman R.J. (1987). Possible neurologic effects of aspartame, a widely used food additive. Environ. Health Perspect..

[92] Nelson D.L., Cox M.M. (2008). Lehninger Principles of Biochemistry.

[93] Rycerz K., Jaworska-Adamu J.E. (2013). Effects of aspartame metabolites on astrocytes and neurons. Folia Neuropathol..

[94] Ardalan M.R., Tabibi H., Attari V.E., Mahdavi A.M. (2017). Nephrotoxic effect of aspartame as an artificial sweetener: A brief review. Iran. J. Kidney Dis..

[95] Debras C., Chazelas E., Sellem L., Porcher R., Druesne-Pecollo N., Esseddik Y., de Edelenyi S.F., Agaësse C., De Sa A., Lutchia R. (2022). Artificial sweeteners and risk of cardiovascular diseases: Results from the prospective NutriNet-SantÃ© cohort. BMJ.

[96] Alwaleedi S.A. (2016). Alterations in antioxidant defense system in hepatic and renal tissues of rats following aspartame intake. J. Appl. Biol. Biotechnol..

[97] Halliwell B., Gutteridge J.M.C. (2015). Free Radicals in Biology and Medicine.

[98] Anbara H., Kian M., Darya G.H., Sheibani M.T. (2022). Long-term intake of aspartame-induced cardiovascular toxicity is reflected in altered histochemical parameters, evokes oxidative stress, and trigger P53-dependent apoptosis in a mouse model. Int. J. Exp. Pathol..

[99] Choudhary A.K., Rathinasamy S., Sundareswaran L. (2014). Role of antioxidant enzymes in oxidative stress and immune response evaluation of aspartame in blood cells of wistar albino rats. Int. Food Res. J..

[100] Griebsch L.V., Theiss E.L., Janitschke D., Erhardt V.K.J., Erhardt T., Haas E.C., Kuppler K.N., Radermacher J., Walzer O., Lauer A.A. (2023). Aspartame and its metabolites cause oxidative stress and mitochondrial and lipid alterations in SH-SY5Y cells. Nutrients.

[101] Maslova E., Strøm M., Olsen S.F., Halldorsson T.I. (2013). Consumption of artificially-sweetened soft drinks in pregnancy and risk of child asthma and allergic rhinitis. PLoS ONE.

[102] Bassett J.K., Milne R.L., English D.R., Giles G.G., Hodge A.M. (2020). Consumption of sugar-sweetened and artificially sweetened soft drinks and risk of cancers not related to obesity. Int. J. Cancer.

[103] Debras C., Chazelas E., Srour B., Druesne-Pecollo N., Esseddik Y., de Edelenyi S.F., Agaësse C., De Sa A., Lutchia R., Gigandet S. (2022). Artificial sweeteners and cancer risk: Results from the NutriNet-Santé population-based cohort study. PLoS Med..

[104] Choudhary A.K., Pretorius E. (2017). Revisiting the safety of aspartame. Nutr. Rev..

[105] Liu L., Zhang P., Wang Y., Cui W., Li D. (2021). The relationship between the use of artificial sweeteners and cancer: A meta-analysis of case-control studies. Food Sci. Nutr..

[106] Guercio B.J., Zhang S., Niedzwiecki D., Li Y., Babic A., Morales-Oyarvide V., Saltz L.B., Mayer R.J., Mowat R.B., Whittom R. (2018). Associations of artificially sweetened beverage intake with disease recurrence and mortality in stage III colon cancer: Results from CALGB 89803 (Alliance). PLoS ONE.

[107] Schiano C., Grimaldi V., Franzese M., Fiorito C., De Nigris F., Donatelli F., Soricelli A., Salvatore M., Napoli C. (2020). Non-nutritional sweeteners effects on endothelial vascular function. Toxicol. Vitr..

[108] Pandurangan M., Enkhtaivan G., Kim D.H. (2016). Cytotoxic effects of aspartame on human cervical carcinoma cells. Toxicol. Res..

[109] Reiner A., Levitz J. (2018). Glutamatergic Signaling in the Central Nervous System: Ionotropic and Metabotropic Receptors in Concert. Neuron.

[110] Polizzi S., Pira E., Ferrara M., Bugiani M., Papaleo A., Albera R., Palmi S. (2002). Neurotoxic effects of aluminium among foundry workers and Alzheimer’s disease. Neurotoxicology.

[111] Olney J.W. (1969). Brain lesions, obesity, and other disturbances in mice treated with monosodium glutamate. Science.

[112] Olney J.W. (1984). Excitotoxic food additives—Relevance of animal studies to human safety. Neurobehav. Toxicol. Teratol..

[113] Choudhary A.K., Lee Y.Y. (2017). Neurophysiological symptoms and aspartame: What is the connection?. Nutr. Neurosci..

[114] Mourad I.M., Noor N.A. (2011). Aspartame (a widely used artificial sweetener) and oxidative stress in the rat cerebral cortex. Int. J. Pharm. Biomed. Sci..

[115] Lipton S.A., Choi Y.-B., Sucher N.J., Chen H.-S.V. (1998). Neuroprotective versus neurodestructive effects of NO-related species. Biofactors.

[116] Yoshikawa T., Naito Y. (2000). What is oxidative stress?. Jpn. Med. Assoc. J..

[117] Park S., Sethi S., Bouret S.G. (2019). Non-nutritive Sweeteners Induce Hypothalamic ER Stress Causing Abnormal Axon Outgrowth. Front. Endocrinol..

[118] Park C.H., Choi S.H., Piao Y., Kim S., Lee Y.J., Kim H.S., Jeong S.J., Rah J.C., Seo J.H., Lee J.H. (2000). Glutamate and aspartate impair memory retention and damage hypothalamic neurons in adult mice. Toxicol. Lett..

[119] Lee J., Chung M.K., Ro J.Y. (2012). Activation of NMDA receptors leads to phosphorylation of TRPV1 S800 by protein kinase C and A-Kinase anchoring protein 150 in rat trigeminal ganglia. Biochem. Biophys. Res. Commun..

[120] Lee J., Saloman J.L., Weiland G., Auh Q.S., Chung M.K., Ro J.Y. (2012). Functional interactions between NMDA receptors and TRPV1 in trigeminal sensory neurons mediate mechanical hyperalgesia in the rat masseter muscle. Pain.

[121] Abdollahi M., Nikfar S., Abdoli N. (2001). Potentiation by nitric oxide synthase inhibitor and calcium channel blocker of aspartame-induced antinociception in the mouse formalin test. Fundam. Clin. Pharmacol..

[122] Errico F., Nisticò R., Palma G., Federici M., Affuso A., Brilli E., Topo E., Centonze D., Bernardi G., Bozzi Y. (2008). Increased levels of d-aspartate in the hippocampus enhance LTP but do not facilitate cognitive flexibility. Mol. Cell. Neurosci..

[123] Council on Scientific Affairs (1985). Aspartame. Review of safety issues. JAMA.

[124] Malkan S. (2022). Aspartame: Decades of Science Point to Serious Health Risks. https://usrtk.org/sweeteners/aspartame_health_risks/.

[125] Millstone E.P., Dawson E. (2019). EFSA’s toxicological assessment of aspartame: Was it even-handedly trying to identify possible unreliable positives and unreliable negatives?. Arch. Public Health.

[126] Spiers P.A., Sabounjian L., Reiner A., Myers D.K., Wurtman J., Schomer D.L. (1998). Aspartame: Neuropsychologic and neurophysiologic evaluation of acute and chronic effects. Am. J. Clin. Nutr..

[127] Solomi L., Rees G.A., Redfern K.M. (2019). The acute effects of the non-nutritive sweeteners aspartame and acesulfame-K in UK diet cola on glycaemic response. Int. J. Food Sci. Nutr..

[128] Torii K., Mimura T., Takasaki Y., Ichimura M. (1986). Dietary aspartame with protein on plasma and brain amino acids, brain monoamines and behavior in rats. Physiol. Behav..

[129] Sharma R.P., Coulombe R.A. (1987). Effects of repeated doses of aspartame on serotonin and its metabolite in various regions of the mouse brain. Food Chem. Toxicol..

[130] Vences-Mejía A., Labra-Ruíz N., Hernández-Martínez N., Dorado-González V., Gómez-Garduño J., Pérez-López I., Nosti-Palacios R., Camacho Carranza R., Espinosa-Aguirre J.J. (2006). The effect of aspartame on rat brain xenobiotic-metabolizing enzymes. Hum. Exp. Toxicol..

[131] Helal E.G., Abdelaziz M.A., Taha N.M., El-Gama M.S. (2019). The influence of acesulfame-k and aspartame on some physiological parameters in male albino rats. Egypt. J. Hosp. Med..

